# Initial overcorrection after surgery for intermittent exotropia in children less than 4 years old: Comparison with older children

**DOI:** 10.1371/journal.pone.0257465

**Published:** 2021-09-23

**Authors:** Jinju Choi, Dong Gyu Choi

**Affiliations:** Department of Ophthalmology, Kangnam Sacred Heart Hospital, Hallym University College of Medicine, Seoul, Korea; National Taiwan University Hospital, TAIWAN

## Abstract

**Purpose:**

While initial overcorrection after exotropia-correcting surgery is widely accepted for a favorable long-term outcome, some have not advocated such overcorrection in younger children owing to concerns regarding rapid deterioration of bifixation ability. This study aimed to evaluate the relationship between initial overcorrection after intermittent exotropia surgery and the surgical outcome in patients aged <4 years.

**Methods:**

In this retrospective study, 391 patients who had undergone surgery for intermittent exotropia were classified into two groups according to the age at surgery: <4 years old (group Y [young], 130 patients) and 4–16 years old (group O [old], 261). The patients were subdivided into three groups according to the angle of deviation at postoperative 1 week: esophoria-tropia (ET) ≥10 prism diopters (PD) (subgroup I), ET 1–9 PD (II), and orthotropia or exophoria-tropia (XT) (III). We compared the surgical outcomes between the two groups and among subgroups; then, we analyzed consecutive esotropia patients.

**Results:**

The mean exodeviation was smaller in the order of subgroup I, II, and III at every postoperative visit (p<0.05) in group Y but showed no difference among subgroups after 2 years in group O. Consecutive esotropia occurred at 1 month, postoperatively, in 6.9% and 2.6% of the patients in groups Y and O (p = 0.133), respectively. However, it persisted in two and one patient in groups Y and O, respectively, until the last visit.

**Conclusion:**

Early overcorrection after intermittent exotropia surgery was a safe and desirable result in terms of motor outcome in children aged under 4 years, as well as for children aged between 4–16 years.

## Introduction

The need for surgery in intermittent exotropia is determined by the state of fusional control, the angle size of deviation, and the age of the patient [1]. The surgical outcomes of intermittent exotropia vary according to the definition of surgical success and the postoperative follow-up period [2–7]. Numerous factors reported in many studies that are likely to affect outcomes of intermittent exotropia surgery include age at onset, the timing of surgery, preoperative angle of deviation, A and V pattern, and amblyopia. However, no agreement has been established except for a postoperative initial overcorrection [8–11]. Although most strabismus surgeries aim to align the eyes as closely as possible, it has been widely accepted that, for intermittent exodeviations, the initial postoperative alignment should be targeted to esodeviation for favorable long-term motor alignment because of a tendency toward postoperative exotropic drift [1,3,6,12–16]. Postoperative diplopia caused by esotropic overcorrection may stimulate the development of fusional vergences and stabilize postoperative alignment [1,17]. However, some authors suggested that intentional overcorrection should be avoided in younger children with an immature visual system because even a small overcorrection may lead to a monofixational esotropia, resulting in deteriorating stereopsis, developing amblyopia, and loss of fusion in this group [17,18] Therefore, we compared the outcomes of intermittent exotropia surgery in children under 4 years of age and children aged between 4–16 years, according to early postoperative deviation, to investigate whether early overcorrection is a safe and desirable surgical goal even for children under 4 years of age.

## Materials and methods

### Subject recruitment

The medical records of consecutive patients under 16 years of age who had undergone surgery for intermittent exotropia with poor control between January 2005 and January 2017 with a postoperative follow-up of ≥6 months were reviewed retrospectively. The control of exodeviation was scaled as good, fair, or poor. Specifically, good, fair, and poor controls were defined as follows: “fusion breaks only after cover testing at distance fixation and resumes rapidly without need for blinking or refixation,” “subject blinks or refixates to control deviation after disruption with cover testing at distance fixation,” “subject breaks spontaneously without any form of fusion disruption or does not spontaneously regain alignment despite blinking or refixation,” respectively [19]. Exclusion criteria were the following: (1) history of strabismus surgery, (2) simultaneous vertical and/or oblique muscle surgery, (3) sensory, paralytic, or restrictive exotropia, (4) other ocular diseases, or (5) systemic anomaly such as a neurologic disorder or a developmental delay. Patients with A or V pattern dissociated vertical deviation (DVD), or oblique muscle overactions not requiring surgical correction were included. This study’s protocol adhered to the Declaration of Helsinki and was approved by the Institutional Review Board of Hallym University Medical Center (approval no. 2020-11-017), and informed consent was waived owing to the retrospective nature of the study.

### Preoperative evaluation

All patients underwent complete ophthalmological examinations before surgery. We recorded the preoperative characteristics, including sex, age at onset of deviation, age at surgery, deviation at a distance and near, presence of amblyopia, vertical deviation, DVD, oblique muscle dysfunction, and stereopsis. Cycloplegic refraction was performed with 1% cyclopentolate hydrochloride (Cyclogyl, Alcon Laboratories Inc., Fort Worth, TX, USA) and 1% tropicamide (Mydriacyl, Alcon Laboratories Inc.). The angle of deviation was primarily determined by the prism and alternating-cover test with accommodative targets both at a distance (6 m) and near (33 cm) with their best optical correction. Amblyopia was defined as a difference of 2 or more lines between the best-corrected visual acuity of each eye. Vertical deviation was defined as 5 prism diopters (PD)-or-more hyper/hypotropia at the primary position. Sensory status was evaluated by the Titmus Stereotest (Stereo Optical Co., Inc., Chicago, IL, USA) and the Worth 4-Dot test at a distance, if possible. The result was recorded as “fusion” when a patient saw 4 dots in the Worth 4-dot test.

### Surgery and postoperative management

Under general anesthesia, bilateral lateral rectus (BLR) muscle recessions or unilateral lateral rectus (ULR) recess-medical rectus resection (referred to as R&R) was performed by a single surgeon (DGC) who had no preference for either procedure. Patients with exotropia <25 PD both at distant and near fixation usually underwent ULR recession. The surgical dosage was based on the angle of distant deviation (Table 1).

**Table 1:** Surgical dosage based on measurement at distance for intermittent exotropia patients.

PD	BLR (mm)	R&R (mm)	ULR (mm)
15	4.0	4.0/3.0	8.0
20	5.0	5.0/4.0	9.0
25	6.0	6.0/5.0	10.0
30	7.0	7.0/5.5	
35	7.5	7.5/6.0	
40	8.0	8.0/6.5	
50	9.0	9.0/7.0	

PD, prism diopters; BLR, bilateral lateral muscle recession; R&R, unilateral lateral rectus recess-medical rectus resection; ULR = unilateral lateral rectus recession.

Distant and near deviations were measured at week 1 and at 1, 3, 6, 12, and 24 months postoperatively and their last visits thereafter. In patients who underwent reoperation, the visit immediately before reoperation was considered as the last follow-up. Patients with diplopia associated with postoperative esotropia were managed with alternating full-time patching until the diplopia resolved.

If esotropia or diplopia did not disappear by 1 month postoperatively, cycloplegic refraction was rechecked, and the residual hyperopia was corrected. If the esotropia persisted for more than 2 months, base-out Fresnel press-on prisms (3M Press-On Optics TM; 3M Health Care, St Paul, MN, USA) were prescribed to allow constant fusion until the esotropia resolved.

### Grouping

Patients were classified into group Y (young) if the age at surgery was under 4 years, and group O (old), if the age at surgery was 4–16 years. In each group, patients were divided into three subgroups based on the initial postoperative deviation at a distance at 1 week: esophoria-tropia (ET) ≥ 10 prism diopters (PD) (subgroup I), ET 1–9 PD (subgroup II), and orthotropia or exophoria-tropia (XT) (subgroup III).

### Outcome measures

The main outcome measure was the surgical outcome according to the age at surgery (between groups Y and O) and according to the deviations at postoperative week 1 (among subgroups I, II, and III). Additionally, we compared consecutive esotropia patients in both groups.

An alignment of 5 PD ET to 10 PD XT at a distance and near fixation was considered surgical success. Consecutive esotropia was defined as an ET > 5 PD on the distance measurement at 1 month from surgery.

### Statistical analysis

Statistical analyses were performed using SPSS version 24.0 (SPSS Inc., Chicago, IL, USA). A comparison of patients’ demographic data between Groups Y and O was performed using the Mann–Whitney U-test, Pearson’s chi-squared test, and Fisher’s exact test. Analysis of variance was used to compare the postoperative deviation and Pearson chi-square test to compare the incidence of consecutive esotropia among subgroups I, II, and III in groups Y and O, respectively. P-values of <0.05 were considered statistically significant. The surgical success rate and incidence of consecutive esotropia were compared using the Pearson’s chi-square test. In the angle of deviation, “minus” and “plus” indicate esodeviation and exodeviation, respectively.

## Results

The demographic characteristics of the enrolled patients are presented in Table 2. A total of 391 patients were included, with 130 patients in Group Y and 261 in Group O. The mean age at surgery was 34.57 ± 8.87 months in group Y and 93.82 ± 31.37 months in group O. The patients’ mean preoperative angle of deviation was 28.95 ± 7.35 PD and 25.52 ± 7.16 at a far distance in groups Y and O, respectively (p<0.05), and 28.18 ± 12.39 and 24.72 ± 9.37 at a near distance (p<0.05), respectively.

**Table 2:** Demographic characteristics of enrolled patients.

	Group Y (n = 130)	Group O (n = 261)	p-value
Sex (male: female)	49:81	123:138	0.715*
Age at surgery (months)	34.57 ± 8.87	93.82 ± 31.37	<0.001†
Preoperative exodeviation (PD)			
at distance	28.95 ± 7.35	25.52 ± 7.16	<0.001†
at near	28.18 ± 12.39	24.72 ± 9.37	<0.001†
Amblyopia [n, (%)]	11 (8.5)	27 (10.3)	0.297‡
Associated strabismus			
DVD [n, (%)]	17 (13.1)	7 (2.7)	1.00‡
Vertical deviation [n, (%)]	19 (14.6)	64 (24.5)	0.251‡
Oblique muscle dysfunction [n, (%)]	31 (23.8)	62 (23.8)	0.769*
Sensory status			
100 arc sec or better in Titmus test [n, (%)]	17/36 (47.2)	203/237 (85.7)	0.656‡
Fusion on the Worth-4-dot test [n, (%)]	11/30 (36.7)	91/222 (41.0)	1.00‡
Postoperative follow-up period (months)	37.72 ± 26.92	36.69 ± 28.97	0.358†

* Pearson chi-squared test,

^†^ Mann–Whitney U test,

^‡^ Fisher’s exact test.

PD = prism diopters, DVD = dissociated vertical deviation.

Vertical deviation = 5 PD or more hypertropia/hypotropia at primary position.

Group Y: Patients under 4 years old, Group O: Patients between 4 and 16 years of age.

Among group Y, 37 patients (28.5%) were classified into subgroups I (ET ≥ 10 PD at postoperative week 1), 41 (31.5%) into subgroup II (ET 1–9 PD), and 52 (40%) into subgroup III (orthotropia or XT). Among group O, 51 (19.5%), 89 (34.1%), and 121 patients (46.4%) were classified into subgroups I, II, and III, respectively. There was no statistically significant difference in the subgroup distribution was between groups Y and O (p = 0.133, Pearson’s chi-square test) (Table 3).

**Table 3:** Angle of deviation at 1 week postoperatively in groups Y and O.

	Group Y (n = 130)	Group O (n = 261)	p-value*
			0.133
Subgroup I (esodeviation ≥ 10 PD)	37 (28.5%)	51 (19.5%)	
Subgroup II (1–9 PD esodeviation)	41 (31.5%)	89 (34.1%)	
Subgroup III (ortho or exodeviation)	52 (40%)	121 (46.4%)	

Group Y: Patients under 4 years of age, Group O: Patients between 4 and 16 years of age.

* Pearson chi-squared test.

**Fig 1:**
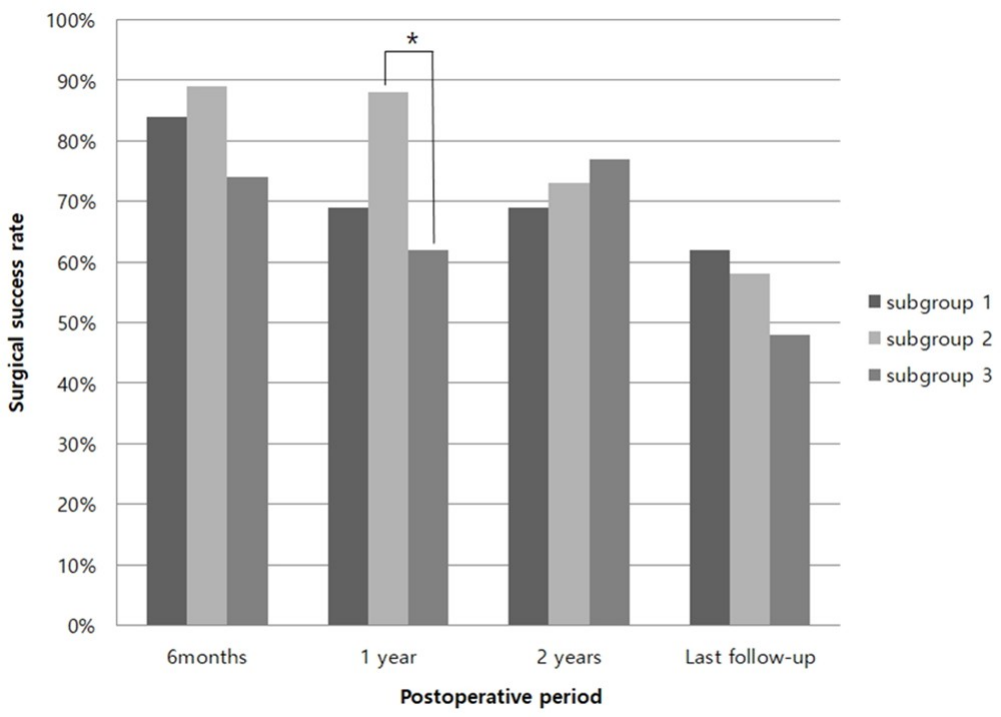
Surgical success rate in group Y (under 4 years old). *p<0.05, Statistically significant (Pearson’s chi-square test). Group Y: patients under 4 years of age, Group O: patients between 4 and 16 years of age Subgroup I: postoperative 1 week esophoria-tropia (ET) ≥ 10 prism diopters (PD), Subgroup II: postoperative 1 week ET 1-9 PD, Subgroup III: postoperative 1 week ortfaotropia or exophoria-tropia (XT).

**Fig 2:**
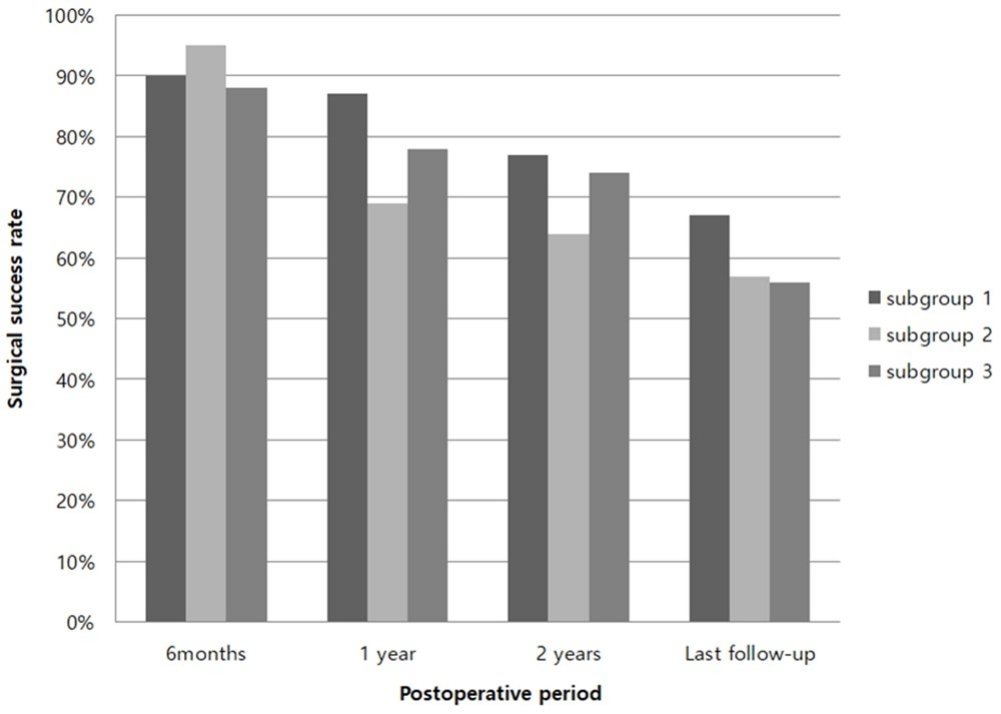
Surgical success rate in group O (4–16 years old). *p<0.05, Statistically significant (Pearson’s chi-square test). Group Y: patients under 4 years of age, Group O: patients between 4 and 16 years of age Subgroup I: postoperative 1 week esophoria-tropia (ET) ≥ 10 prism diopters (PD), Subgroup II: postoperative 1 week ET 1-9 PD, Subgroup III: postoperative 1 week orthotropia or exophoria-tropia (XT).

In group Y, the larger the initial esodeviation (in order group I, II, and III), the smaller the mean exodeviation, significantly at every visit (from postoperative 1 month to the last follow-up) (p<0.05) (Table 4). In group O, the mean exodeviation was also significantly smaller in groups I, II, and III until 1 year postoperatively (p<0.05, respectively). However, no difference was found among subgroups after 2 years (Table 5). In group Y, the surgical success rate was significantly higher in group II than in group III only at 1 year postoperatively. However, there was no difference among the subgroups at any other visit (Fig 1). Group O showed no statistical difference among subgroups I, II, and III in the surgical success rate at every visit postoperatively (p>0.05) (Fig 2).

**Table 4:** Postoperative mean angle of deviation at distance in group Y (under 4 years of age) (N = 130).

	Subgroup I	Subgroup II	Subgroup III	p-value*
1 month (n = 130)	-2.03 ± 3.	0.15 ± 2.07	4.15 ± 9.33	<0.001
3 months (n = 130)	-1.20 ± 4.30	1.82 ± 5.11	5.04 ± 9.83	0.001
6 months (n = 130)	-1.16 ± 4.64	2.16 ± 6.39	7.32 ± 10.31	<0.001
1 year (n = 102)	-1.37 ± 6.60	2.44 ± 5.	9.21 ± 11.87	<0.001
2 years (n = 89)	0.97 ± 8.58	7.10 ± 8.67	7.53 ± 10.03	0.011
Last follow-up	6.24±11.71	10.12±10.18	12.17±11.08	0.045

* One-way analysis of variance (ANOVA).

Subgroup I: Postoperative 1 week esophoria- tropia (ET) ≥ 10 prism diopters (PD), Subgroup II: Postoperative 1 week ET 1–9 PD, Subgroup III: Postoperative 1 week orthotropia or exophoria-tropia (XT).

Positive numbers represent exodeviation; negative numbers, esodeviation.

**Table 5:** Postoperative mean angle of deviation at distance in group O (4–16 years of age) (N = 261).

	Subgroup I	Subgroup II	Subgroup III	p-value*
1 month (n = 261)	-1.00 ± 3.29	0.39 ± 2.64	3.04 ± 4.79	<0.001
3 months (n = 261)	-0.09 ± 4.19	1.76 ± 4.04	4.68 ± 5.98	<0.001
6 months (n = 261)	1.27 ± 4.85	2.99 ± 4.71	5.59 ± 5.84	<0.001
1 year (n = 215)	2.64 ± 7.70	5.09 ± 7.13	6.54 ± 6.43	0.008
2 years (n = 185)	5.30 ± 7.23	7.26 ± 8.84	7.74 ± 7.19	0.311
Last follow-up	6.61 ± 12.04	10.01 ± 10.29	10.05 ± 9.22	0.100

* One-way analysis of variance (ANOVA).

Subgroup I: Postoperative 1 week esophoria- tropia (ET) ≥ 10 prism diopters (PD), Subgroup II: Postoperative 1 week ET 1–9 PD, Subgroup III: Postoperative 1 week orthotropia or exophoria-tropia (XT).

Positive numbers represent exodeviation; negative numbers, esodeviation.

Table 6 shows the incidence of good stereopsis (= 100 arcsec or better) and fusion on the Woth-4-dot test according to the subgroups of groups Y and O. As all patients in group Y were aged <4 years, it was not easy to obtain the results of the sensory status, as measured by the Titmus and Worth-4-dot tests. Moreover, the number of patients who had the records of those tests was not sufficient to perform a statistical analysis. However, the number of patients with good stereopsis and fusion increased in all subgroups postoperatively, even in subgroup I of group Y. No patient in groups Y and O demonstrated the postoperative deterioration in the Titmus and Worth-4-dot tests.

**Table 6:** Preoperative and postoperative sensory status measurements by the Titmus and Worth-4-dot tests.

	Good stereopsis* in the Titmus Stereotest		Fusion on the Worth-4-dot test	
	preoperatively	postoperatively	preoperatively	postoperatively
Group Y				
Subgroup I	5/9 (55.6%)	18/21 (85.7%)	5/12 (41.7%)	12/18 (66.7%)
Subgroup II	6/11 (54.5%)	15/16 (93.8%)	3/10 (30.0%)	9/13 (69.2%)
Subgroup III	6/16 (37.5%)	18/21 (85.7%)	3/8 (37.5%)	11/17 (64.7%)
Group O				
Subgroup I	40/49 (81.6%)	33/33 (100%)	13/47 (27.7%)	16/29 (55.2%)
Subgroup II	78/88 (88.6%)	65/67 (97.0%)	37/86 (43.0%)	37/61 (60.7%)
Subgroup III	102/118 (86.4%)	94/98 (95.9%)	50/116 (43.1%)	61/89 (68.5%)

*100 arcsec or better.

Group Y: Patients under 4 years old, Group O: Patients between 4 and 16 years of age.

Subgroup I: Postoperative 1 week esophoria- tropia (ET) ≥ 10 prism diopters (PD), Subgroup II: Postoperative 1 week ET 1–9 PD, Subgroup III: Postoperative 1 week orthotropia or exophoria-tropia (XT).

Seven (5.38%) and 18 patients (6.90%) patients in groups Y and O, respectively, underwent reoperation for recurrent exotropia within 2 years from the first operation. All patients in group Y who underwent reoperation were included in subgroup III. In group O, there were 4, 4, and 10 patients who underwent reoperation in subgroups I, II, III, respectively.

Consecutive esotropia occurred in 9 (6.9%) patients in group Y (8, 1, and 0 patients in subgroups I, II, and III, respectively) and in 7 (2.6%) patients in group O (5, 2, and 0 patients in subgroups I, II, and III, respectively) at 1 month postoperatively; the difference between groups Y and O was not statistically significant (p = 0.695). However, it only persisted in 2 patients in group Y (2 in I) and 1 in group O until the last visit. Due to poor cooperation and visual immaturity, the preoperative sensory status (as measured by the Titmus and Worth-4-dot tests) was not obtained in most consecutive esotropia patients in group Y. Therefore, comparing the postoperative deterioration of sensory status in consecutive esotropia between groups Y and O was not possible. However, of the patients with consecutive esotropia (9 and 7 in groups Y and O, respectively), 66.7% (4/6) in group Y reported 100 arcsec or better in the Titmus Stereotest and 50% (3/6) reported fusion on the Worth-4-dot test, while these values were reported in 85.7% (6/7) and 57.1% (4/7) of patients in group O, respectively, which indicated comparable results between the 2 groups.

Preoperatively, amblyopia was present in 11 patients (8.5%) in group Y and 27 (10.3%) in group O, and anisometropia was responsible for amblyopia in all patients. Amblyopia improved after surgery in most patients of both groups (9/11 and 25/27 patients in groups Y and O, respectively) through continuous occlusion therapy. Furthermore, amblyopia did not develop postoperatively in patients who did not have amblyopia preoperatively, regardless of postoperative alignment (overcorrected or not). Of 11 patients with amblyopia in group Y, 3 were included in subgroup 1, 5 in subgroup 2, and 3 in subgroup 3. Amblyopia improved postoperatively, except for 1 patient in subgroup 2 and 1 in subgroup 3. In contrast, of 27 patients with amblyopia in group O, 4 were included in subgroup 1, 5 in subgroup 2, and 18 in subgroup 3. Only 2 patients did not improve until the last follow-up in subgroup 3. Although performing statistical analysis was not possible due to the small number of patients included, we did not observe any remarkable trend in amblyopia development in the postoperatively overcorrected groups (subgroups 1 and 2) compared to the non-overcorrected group (subgroup 3).

## Discussion

As some surgeons have not advocated intentional initial overcorrection after surgery for intermittent exotropia in younger children owing to concerns about the rapid loss of bifixation ability, we compared the surgical outcomes of children under 4 and 4–16 years of age according to the early postoperative deviation. We found that initial overcorrection is a safe and desirable surgical goal even in children under 4 years of age, showing outcomes similar to those of children aged between 4–16 years.

Oh and Hwang [15] concluded that postoperative day 1 overcorrection was the only factor to guarantee a successful long-term outcome after exotropia surgery. Lee and Lee [20] also suggested that the alignment at postoperative day 1 can be a predictive factor of the surgical outcome in intermittent exotropia and that overcorrection of 11–20 PD following BLR surgery and 1–10 PD following R&R can lead to good results. Scott and colleagues [3] suggested 4 PD to 14 PD of overcorrection in BLR recession, while McNeer [13] recommended the overcorrection of 0 PD to 10 PD. When the R&R approach is taken, Souza-Dias and Uesugui [14] suggested 5 PD to 10 PD of esotropia. Raab and Parks [12] found good outcomes for BLR recession with overcorrection of 0 to 10 PD but even better outcomes with overcorrection of 10 to 20 PD.

However, Choi et al. [6] reported that although initial overcorrection after intermittent exotropia surgery may be associated with a lower probability of recurrence within 2 years from surgery, it cannot predict long-term motor outcomes. Moreover, Ruttum [10] found that an initial alignment within the range of orthotropia to 9 PD of esotropia 1 to 3 days after the operation does not guarantee a good final outcome. Leow et al. [21] also reported a good correlation between ocular alignment at 1 week and 6 months postoperatively. However, the success rate, defined as an ocular deviation within 10 PD of orthophoria, appears to be unaffected by initial ocular alignment. Nevertheless, despite these controversies, postoperative initial overcorrection has been known as the only predictive factor of long-term motor results of the surgical treatment.

However, von Noorden & Campos [1] advised that initial overcorrection must be avoided under all circumstances in visually immature children owing to the disastrous consequences of a small angle esotropia in this age group. Edelman et al. [17] found that 5 of 24 children who developed consecutive esotropia after surgery for exotropia before 4 years of age became amblyopic. Even when surgery was delayed until 4–6 years of age, amblyopia still occurred in 3 out of 39 patients. They concluded that patients under 6 years of age are at risk of developing amblyopia and totally losing stereoacuity as a response to overcorrection (consecutive esodeviation). Jampolsky [18] also advised delaying surgery in visually immature infants to avoid overcorrection.

However, Richard and Parks [22] reported that there was no significant difference in results between early and late surgery. Moreover, Pratt-Johnson et al. [23] found better surgical outcomes when surgery was performed under 4 years of age.

In this study, 6.9% of patients under 4 years of age and 2.6% of patients aged between 4–16 years showed consecutive esotropia at 1 month from surgery, but only 2 patients and 1 patient showed persistent esotropia at the last follow-up visit. The incidence of consecutive esotropia in children under 4 years of age was higher than in children aged >4 years; however, the difference was not statistically significant.

Cho and Lee [24] found that the consecutive esotropia persisted at 1 year after surgery in 4 of 37 (10.8%) patients younger than 4 years of age and in 19 of 276 (6.9%) children aged 4 years or older (p = 0.39), which is consistent with the results of this study.

There was a concern regarding the induction of esotropia, rendering patients either diplopic or unable to fuse, even if only for a short term, as it could significantly increase the risk of binocularity loss. Of 14 patients who underwent ULR in group Y, consecutive ET (defined as an ET > 5 PD at 1 month from surgery) occurred in 1 patient. None of the ULR patients in group Y, including the patient with consecutive ET, showed either abnormal head posture or abduction limitation. In contrast, none of the 72 patients who underwent ULR in group O showed consecutive ET after 1 month or abnormal head posture. However, 1 patient in this group showed abduction limitation, which disappeared after 3 months. Similar results regarding abduction limitation following ULR recession were reported by Kim et al. [25].

This study has some limitations. First, since this study was conducted retrospectively, we might have included different types of intermittent exotropia and various surgical methods. Thus, there might have been a minor selection bias. Second, the statistical analysis of the sensory status of patients with consecutive esotropia in both groups could not be performed because the incidence of consecutive esotropia was low. Moreover, the Titmus and Worth-4-dot tests could not be performed for most patients in group Y (<4 years at surgery) due to a lack of patient cooperation. Finally, the preoperative exodeviation in group Y was significantly larger than in group O, which could have caused some selection bias. The surgeon might have decided to perform surgery in patients under 4 years of age when the exodeviation angles were larger compared to that in older children. We mainly analyzed the surgical outcome according to the initial postoperative alignment, especially overcorrection. It may be worthwhile to compare the postoperative results between three subgroups based on the initial postoperative deviation. Therefore, further prospective, randomized, and controlled studies with a comparison of sensory status including large numbers of patients are recommended.

In conclusion, early intentional overcorrection after intermittent exotropia surgery was safe concerning the motor outcome in both children aged between 4–16 years and children under 4 years of age. The consecutive esotropia in children under 4 years of age was not significantly higher than that in children over 4 years of age when observed for more than 1 year. Therefore, these results should be considered when determining the time and the target angle of surgery for intermittent exotropia.
